# The Gastro-Circadian Metabolic Axis: A Comprehensive Framework for Chronotherapy in Gastroenterology

**DOI:** 10.7759/cureus.110724

**Published:** 2026-06-12

**Authors:** Mohammed Alkhaldi, Alejandra Felix Vicente, Victoria Fansey, Rachel Melissa Salins, Ahmad Mahmoo, Mariam Alamgir, Ann Maria Dominic, Mohamed Izzeldin S Siddig, Cheree Suk, Quratul Ain Haider, Jocelyn N Wensel, Manju Rai

**Affiliations:** 1 Internal Medicine, University College Dublin, Dublin, GBR; 2 Internal Medicine, Universidad de Sonora, Hospital Infantil del Estado de Sonora, Sonora, MEX; 3 General Medicine, University of Nis, Nis, SRB; 4 General Medicine, Kasturba Medical College, Mangalore, IND; 5 General Surgery, Xinjiang Medical University, Xinjiang, CHN; 6 Medicine, Washington State University, Washington, USA; 7 Internal Medicine, St. John’s Medical College Hospital, Bangalore, IND; 8 Internal Medicine, South Tees University Hospital, Middlesborough, GBR; 9 Internal Medicine, Kyungpook National University School of Medicine, Daegu, KOR; 10 Internal Medicine, Poonch Medical College, Rawalakot, PAK; 11 Internal Medicine, International University of Health Sciences, Basseterre, KNA; 12 Biotechnology, Shri Venkateshwara University, Gajraula, IND

**Keywords:** chrononutrition, chronotherapy, circadian rhythms, gastro-circadian metabolic axis, gastrointestinal disorders, gut microbiome, metabolic homeostasis, time-restricted feeding

## Abstract

Circadian rhythms exert fundamental control over gastrointestinal and metabolic physiology, governing 24-hour patterns of motility, secretion, nutrient absorption, microbial activity, immune regulation, and hepatic metabolism. Accumulating evidence indicates that the gastrointestinal tract is not an isolated system but is tightly integrated with systemic metabolic and neuroendocrine networks, forming a coordinated gastro-circadian metabolic axis (GCMA). This axis links molecular clocks in the gut, liver, adipose tissue, and skeletal muscle with rhythmic inputs from the gut microbiome, feeding-fasting cycles, autonomic signaling, and enteroendocrine mediators. Disruption of circadian alignment, through shift work, sleep deprivation, irregular meal timing, or nocturnal light exposure, leads to desynchronization between central and peripheral clocks, promoting inflammation, impaired epithelial barrier function, dysbiosis, altered bile acid signaling, insulin resistance, and disturbed energy homeostasis. These mechanisms contribute to a wide spectrum of gastrointestinal disorders, including gastroesophageal reflux disease, functional dyspepsia, irritable bowel syndrome, metabolic dysfunction-associated steatotic liver disease, inflammatory bowel disease, and potentially gastrointestinal malignancies. This review synthesizes molecular, translational, and clinical evidence to position the GCMA as a unifying framework for understanding circadian influences on digestive and metabolic disease. Importantly, it highlights emerging therapeutic opportunities in chronotherapy, including time-optimized pharmacotherapy, chrononutrition, and microbiota-targeted interventions. While current translation is limited by interindividual chronotype variability and heterogeneous clinical evidence, advances in wearable circadian monitoring, multi-omics profiling, and computational modeling offer promising avenues for precision implementation. Integrating GCMA principles into clinical practice may improve disease outcomes and establish circadian alignment as a cornerstone of preventive and therapeutic gastroenterology.

## Introduction and background

Circadian biology has emerged as a fundamental organizing principle of human physiology, influencing virtually every organ system through highly conserved 24-hour oscillations in gene expression, hormone secretion, metabolism, and cellular homeostasis [1,2]. These rhythms are generated by an endogenous molecular clock and synchronized by environmental cues, primarily light-dark cycles, feeding-fasting patterns, activity-rest cycles, and social schedules. With daily fluctuations controlling motility, gastric acid secretion, digestive enzyme release, intestinal permeability, and interactions with the gut microbiome, the gastrointestinal (GI) tract is one of the body's most rhythmically dynamic systems [3]. When these rhythms are disturbed, digestive and metabolic homeostasis can be severely disrupted, placing individuals at risk for a variety of GI and systemic disorders [3,4].

Advances in chronobiology, microbiome science, and metabolic research have converged to reveal an intricate, multidirectional relationship between the circadian clock and GI physiology. We propose the gastro-circadian metabolic axis (GCMA), a conceptual framework that unifies the rhythmic activities of the gut microbiota, enteroendocrine signaling, autonomic neural pathways, and systemic metabolic responses with molecular clocks found in the gut, liver, and metabolic organs. The novelty of the GCMA framework lies in its integration of circadian biology, GI physiology, microbiome dynamics, and systemic metabolic regulation into a single conceptual model. While previous studies have examined individual components of these interactions, such as the gut-liver axis, gut-brain axis, or circadian regulation of metabolism, the GCMA provides a broader systems-level perspective that emphasizes their reciprocal and time-dependent coordination [1,2]. By highlighting these interconnected pathways, the framework offers a unifying foundation for understanding disease pathogenesis and developing circadian-informed diagnostic and therapeutic strategies in gastroenterology. Disruptions at any node in this network through altered feeding schedules, shift work, sleep loss, or dysbiosis propagate along the axis, triggering inflammatory, metabolic, and maladaptive neurohormonal responses [5].

The increasing clinical significance of this framework has generated interest in chronotherapy, which involves strategically timing interventions to synchronize with biological rhythms and optimize therapeutic outcomes. Chronotherapy has already been beneficial across fields such as oncology, endocrinology, and rheumatology, where the timing of drug administration can affect pharmacokinetics, pharmacodynamics, efficacy, and toxicity [6]. In gastroenterology, recent evidence suggests that analogous principles are relevant to acid-suppressive therapy, nutritional interventions, and potentially immunomodulation. For instance, gastric acid secretion, motility patterns, bile acid turnover, and mucosal immune activity all exhibit circadian variation, meaning that the effectiveness of treatment may change throughout the day [3,7]. Additionally, feeding timing, irrespective of caloric intake, has demonstrated the ability to synchronize peripheral GI and hepatic circadian rhythms while altering gut microbial composition and metabolic outputs, underscoring its potential as a non-pharmacological chronotherapeutic intervention [5,8].

Despite growing evidence supporting these concepts, gastroenterology practice does not typically employ circadian principles. Clinical histories rarely evaluate sleep patterns or chronotype; medication regimens frequently overlook physiological rhythms; dietary counseling emphasizes what to consume rather than when to consume it; and clinical trials infrequently report or account for diurnal variations. This disparity between mechanistic understanding and clinical application highlights the need for a holistic, integrative framework to guide research and therapeutic implementation.

This review aims to synthesize current evidence on the molecular, microbial, metabolic, and clinical dimensions of circadian regulation in GI health and disease. We introduce the GCMA as an integrative framework linking GI physiology, microbiome rhythmicity, and systemic metabolic regulation. Furthermore, we examine the role of chronodisruption in common GI disorders, evaluate emerging chronotherapeutic strategies, and highlight key research priorities necessary for the development of circadian-informed precision gastroenterology.

## Review

Methodology

Literature Search Strategy

A comprehensive and structured literature search was conducted to identify relevant studies examining the relationship between circadian rhythms, GI physiology, metabolic regulation, gut microbiome rhythmicity, sleep-circadian interactions, and chronotherapeutic interventions. Electronic databases, including PubMed/MEDLINE, Scopus, Web of Science, and the Cochrane Library, were systematically searched to retrieve relevant literature published between January 2006 and December 2025. These databases were selected to ensure broad coverage of biomedical, clinical, and translational research relevant to circadian biology, sleep medicine, gastroenterology, and metabolic science.

The search strategy incorporated combinations of Medical Subject Headings (MeSH) terms and free-text keywords to maximize both sensitivity and specificity. Key MeSH terms and search keywords included “Circadian Rhythm,” “Biological Clocks,” “Chronotherapy,” “Sleep-Wake Disorders,” “Gastrointestinal Diseases,” “Gastrointestinal Microbiome,” “Metabolism,” “Chrononutrition,” “Circadian Misalignment,” “Gut-Liver Axis,” and “Metabolic Diseases.” These terms were combined using Boolean operators such as AND, OR, and NOT to refine search results and capture relevant studies examining mechanistic, translational, and clinical aspects of circadian-GI interactions. In addition to electronic database searches, the reference lists of relevant review articles and original studies were manually screened to identify additional eligible publications that may not have been captured in the primary search. The detailed literature search strategy across databases is summarized in Table 1.

**Table 1:** Structured literature search strategy across electronic databases MeSH, Medical Subject Headings

Database	Time frame	Search terms (MeSH and keywords)	Search strategy (Boolean operators)	Filters applied	Number of records retrieved
PubMed/MEDLINE	January 2006 - December 2025	“Circadian Rhythm”, “Biological Clocks”, “Chronotherapy”, “Sleep Wake Disorders”, “Gastrointestinal Diseases”, “Gastrointestinal Microbiome”, “Metabolism”, “Chrononutrition”, “Circadian Misalignment”, “Gut-Liver Axis”, “Metabolic Diseases”	(“Circadian Rhythm” OR “Biological Clocks”) AND (“Gastrointestinal Diseases” OR “Gut Microbiome” OR “Metabolism”) AND (“Chronotherapy” OR “Chrononutrition” OR “Circadian Misalignment”)	English language, Humans & Animals, Peer-reviewed articles	740
Scopus	January 2006 - December 2025	Same as above (adapted to database indexing terms)	(circadian AND gastrointestinal AND metabolism) AND (chronotherapy OR chrononutrition)	English language, Article/Review	321
Web of Science	January 2006 - December 2025	Same keywords adapted to database	TS=(circadian AND gut AND metabolism) AND TS=(chronotherapy OR microbiome)	English language, Article/Review	193
Cochrane Library	January 2006 - December 2025	“Circadian Rhythm”, “Chronotherapy”, “Gastrointestinal Diseases”	circadian AND gastrointestinal AND chronotherapy	Trials and Reviews, English	53
Manual Search	Not restricted	Reference lists of relevant reviews and original articles	Hand-searching of citations from included studies	Relevant peer-reviewed sources only	54

Study Selection and Eligibility Criteria

Studies were considered eligible for inclusion if they were published in peer-reviewed journals between 2006 and 2025 and were available in English. The language restriction was applied to ensure consistency in study appraisal and data interpretation and to focus on literature indexed in major international biomedical databases. However, we acknowledge that relevant circadian biology and chronotherapy research may also exist in other languages, particularly from countries with strong chronobiology research programs such as China, Japan, and Germany. No formal search of non-English databases was performed; therefore, the possibility that related conceptual frameworks or region-specific models analogous to the proposed GCMA have been described in non-English literature cannot be excluded.

Eligible studies included human clinical studies, randomized controlled trials, observational studies, translational research, experimental animal studies, and mechanistic investigations that examined circadian regulation of GI physiology, gut microbiome rhythmicity, metabolic homeostasis, sleep-circadian interactions, or chronotherapy. Studies were selected based on their relevance to circadian biology and their contribution to understanding the physiological, molecular, clinical, or therapeutic implications of circadian rhythms in GI and metabolic health.

Studies were excluded if they were published prior to 2006 or written in languages other than English. Editorials, opinion articles, commentaries, and non-peer-reviewed sources lacking substantive mechanistic or clinical insights were also excluded. Additionally, studies unrelated to circadian biology, sleep-circadian regulation, GI physiology, microbiome rhythmicity, or metabolic outcomes were not considered. Duplicate publications and studies presenting redundant data were carefully screened and excluded to ensure the inclusion of unique and relevant evidence. The detailed inclusion and exclusion criteria applied in this review are summarized in Table 2.

**Table 2:** Inclusion and exclusion criteria for study selection GI, gastrointestinal

Criteria type	Details
Inclusion criteria	• Studies published between January 2006 and December 2025 • Articles published in peer-reviewed journals • Studies available in the English language • Human clinical studies (randomized controlled trials, observational studies) • Experimental animal studies and mechanistic research • Studies evaluating circadian rhythms in GI physiology • Studies assessing gut microbiome rhythmicity • Studies exploring metabolic homeostasis and circadian regulation • Studies addressing sleep-circadian interactions • Studies investigating chronotherapy or time-based interventions
Exclusion criteria	• Studies published before January 2006 • Non-English language publications • Editorials, commentaries, and opinion articles without substantial mechanistic or clinical insights • Non-peer-reviewed sources • Studies unrelated to circadian biology or GI/metabolic outcomes • Duplicate publications or studies with redundant data

Data Extraction and Synthesis

Relevant data from eligible studies were systematically reviewed and qualitatively synthesized. Information extracted from each study included study design, population characteristics, experimental model, circadian exposure or intervention, GI or metabolic outcomes, and key mechanistic or clinical findings. Emphasis was placed on identifying converging evidence across molecular, experimental, translational, and clinical domains to develop an integrative and clinically relevant conceptual framework describing the GCMA.

Given the heterogeneity in study designs, populations, methodologies, and outcome measures, a narrative synthesis approach was adopted rather than a quantitative meta-analysis. Studies were organized and synthesized according to major thematic domains, including molecular circadian mechanisms, gut microbiome rhythmicity, metabolic integration, chronodisruption and GI disorders, and chronotherapeutic interventions. This thematic synthesis enabled the integration of mechanistic insights with clinical implications to provide a comprehensive understanding of circadian influences on GI and metabolic health.

**Figure 1:**
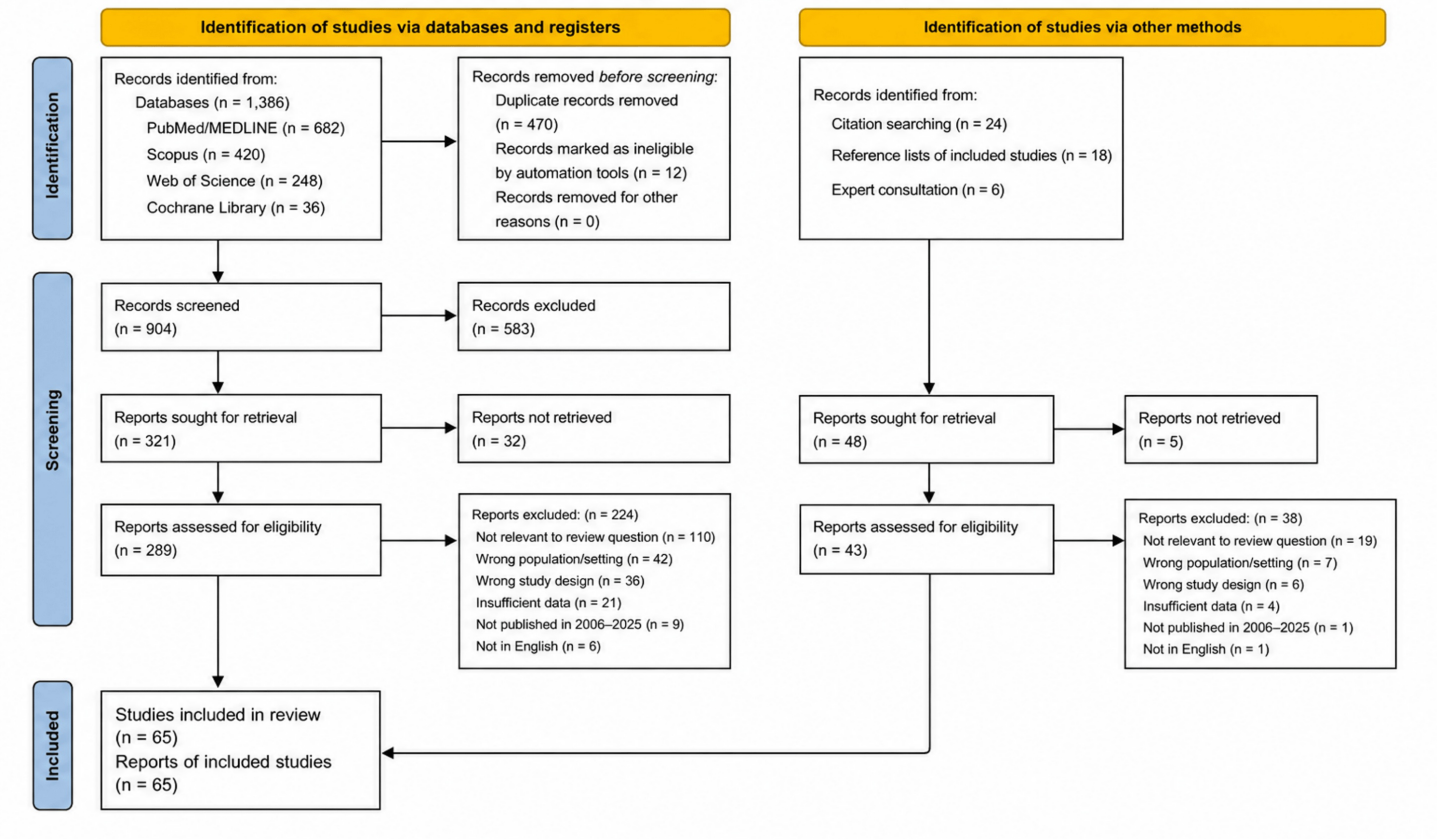
PRISMA 2020 flow diagram illustrating the identification, screening, eligibility assessment, and inclusion of studies considered in this narrative review PRISMA, Preferred Reporting Items for Systematic Reviews and Meta-Analyses

Figure 1 presents a flow diagram illustrating the literature identification, screening, eligibility assessment, and inclusion process for studies considered in this review. Although elements of the Preferred Reporting Items for Systematic Reviews and Meta-Analyses (PRISMA) framework were applied to enhance transparency in literature identification and selection, this review was designed as a narrative synthesis rather than a formal systematic review. Consequently, a standardized risk-of-bias assessment or formal methodological quality appraisal (e.g., the Cochrane Risk of Bias tool or Newcastle-Ottawa Scale) was not performed. Nevertheless, study quality was considered qualitatively during evidence synthesis by evaluating study design, sample size, methodological rigor, consistency of findings, and relevance to the review objectives.

The circadian system: molecular and physiological basis

The circadian system is an evolutionarily conserved timekeeping network that enables organisms to anticipate and adapt to predictable environmental changes over the 24-hour day. Through coordinated regulation of gene expression, metabolism, hormone secretion, and cellular function, circadian clocks synchronize physiological processes with external cues such as light-dark cycles and feeding-fasting patterns. At the molecular level, circadian rhythmicity is generated by a tightly regulated transcriptional-translational feedback loop in which the core transcription factors CLOCK and BMAL1 heterodimerize and bind to E-box (enhancer box) elements (5′-CACGTG-3′) located in the promoter regions of target genes [9]. This binding drives rhythmic transcription of clock-controlled genes, including Period (PER1-3) and Cryptochrome (CRY1-2), as well as a broad array of downstream circadian-regulated genes. The PER and CRY proteins subsequently accumulate in the cytoplasm, undergo post-translational modifications, and translocate back into the nucleus, where they inhibit CLOCK-BMAL1-mediated transcription, thereby completing the negative feedback loop.

Importantly, CLOCK and BMAL1 are not directly regulated by cytoplasmic proteins; rather, cytoplasmic clock gene products exert their regulatory effects through modulation of E-box-mediated transcriptional activity. Genome-wide analyses have identified over 300 E-box-regulated genes associated with circadian processes in the suprachiasmatic nucleus (SCN) and peripheral tissues, underscoring the extensive transcriptional control exerted by this mechanism. This hierarchical, E-box-dependent regulation enables precise temporal coordination of physiological and metabolic pathways across organ systems. Additionally, secondary feedback loops, such as those involving nuclear receptors REV-ERBα/β and RORα/γ, enhance the stability of the rhythm and connect circadian timing to metabolic gene networks [10].

Central and Peripheral Circadian Oscillators

The circadian system is built on a hierarchy, with the SCN of the anterior hypothalamus acting as the master pacemaker. The SCN receives photic signals from retinal ganglion cells that express melanopsin and uses neural, hormonal, and behavioral pathways to keep peripheral clocks in sync [11]. Peripheral oscillators exist throughout the body, including in the GI tract, liver, pancreas, adipose tissue, and skeletal muscle. They maintain their own rhythmicity while also being able to respond to cues that differ from those that reset the SCN [12]. In addition to direct neural and autonomic signaling from the SCN, humoral mediators play a critical role in coordinating circadian timing across organ systems. Among these, melatonin, secreted by the pineal gland in a circadian manner under SCN control, serves as a systemic synchronizing signal for peripheral oscillators. Acting through melatonin receptors (MT1 and MT2) expressed in multiple tissues, melatonin conveys temporal information that facilitates the alignment of peripheral clocks with the external light-dark cycle. Importantly, this entrainment mechanism operates partly independently of direct SCN neuronal output, providing an additional endocrine pathway for circadian coordination. Through this dual mode of regulation, central neural signaling and peripheral hormonal synchronization, the circadian system maintains coherence across diverse physiological systems. Temperature and glucocorticoid rhythms may modify peripheral oscillators independently of light signals [13].

Gastrointestinal Tissue-Specific Rhythmicity

The GI tract manifests strong circadian rhythms in many of its physiological processes. Gastric acid secretion exhibits a diurnal rhythm, characterized by nocturnal peaks influenced by vagal activity and histaminergic pathways, establishing a mechanistic foundation for timing-sensitive presentations of gastroesophageal reflux disease (GERD) and dyspepsia [14]. Intestinal motility exhibits a comparable rhythmic structure: migrating motor complexes decrease during sleep and increase in the early daytime, resulting in consistent daily bowel movement patterns [15]. The secretion of pancreatic enzymes, the synthesis of bile acids, and the motility of the gallbladder also fluctuate throughout the 24-hour cycle, influenced by both SCN-derived autonomic signals and local tissue clocks [16].

Circadian regulation affects epithelial turnover, tight-junction dynamics, and innate immune responses at the mucosal level. Intestinal epithelial cells exhibit diurnal variations in proliferation and apoptosis, with maximal renewal occurring during the resting phase in both humans and rodents [17]. Clock disruption compromises barrier integrity by modifying tight-junction protein expression (e.g., claudins and occludin), thereby enhancing vulnerability to luminal antigens, dysbiosis, and inflammation [18]. Circadian modulation of immune cell trafficking and cytokine expression also affects the tone of the mucosal immune system, contributing to fluctuations in inflammatory responses throughout the day [19].

Hormonal and Neuronal Pathways in Gastro-Circadian Interactions

Hormonal mediators, including cortisol, melatonin, ghrelin, glucagon-like peptide-1 (GLP-1), and insulin, play critical roles in coordinating circadian regulation of GI and metabolic physiology. Cortisol exhibits a pronounced early-morning peak that synchronizes peripheral clocks and modulates glucose metabolism and gastric motility. Rising nocturnally under SCN control, melatonin contributes to the regulation of GI motility, mucosal defense, and antioxidant activity [20]. Meal-induced hormones such as GLP-1 and glucose-dependent insulinotropic polypeptide (GIP) also exhibit circadian variation, with enhanced insulinotropic effects during the daytime, aligning nutrient handling with metabolic efficiency [21].

Neuronal pathways add further layers of integration. The autonomic nervous system transmits SCN output to the gut, where it governs blood flow, motility, and secretion. Vagal signaling links the gut to central circadian circuits and is involved in both receiving and transmitting information. Enteric neurons have their own clocks that regulate the release of neurotransmitters and peristaltic reflexes. This suggests that they can control local rhythms even in the absence of central input [22].

This complex interaction between molecular clocks, neuroendocrine signaling, and tissue-specific rhythmicity creates a highly coordinated 24-hour system that controls metabolic and digestive homeostasis. Disruptions at any level can impair physiological function, forming the basis for the GCMA concept.

The gut microbiome and circadian rhythmicity

The gut microbiome exhibits significant circadian rhythmicity in its composition and metabolic processes. Early sequencing-based studies demonstrated diurnal fluctuations in bacterial abundance, with Firmicutes generally increasing during feeding intervals and Bacteroidetes prevailing during fasting periods. These compositional changes are accompanied by fluctuations in microbial metabolites such as short-chain fatty acids (SCFAs), secondary bile acids, and tryptophan derivatives, all of which vary according to the time of day and impact host physiology [23]. The initial evidence of microbiome rhythmicity was derived from murine models subjected to regulated feeding cycles, wherein Leone et al. demonstrated that limiting feeding to the active phase reinstated rhythmic microbial patterns and enhanced metabolic outcomes in obese mice. This study included 40 mice that were randomly assigned to either ad libitum or time-restricted feeding. The time-restricted feeding group showed better insulin sensitivity and reduced hepatic fat accumulation, but these results may not be applicable to humans [24].

Additionally, feeding-fasting cycles act as the dominant environmental time cue (zeitgeber) for the liver and intestinal clocks, synchronizing peripheral circadian rhythms with nutrient availability and metabolic demands. Human studies have confirmed these findings. In a controlled trial involving 10 healthy adults subjected to 48 hours of regular versus irregular meal timing, Kaczmarek et al. demonstrated disturbances in SCFA oscillations and microbial α-diversity during irregular feeding. The limited sample size and brief duration restrict generalizability; however, the findings offer preliminary evidence that meal timing significantly influences microbiome rhythmicity [25]. The timing of sleep and exposure to light also affect microbial dynamics. In a crossover study involving 14 adults experiencing circadian misalignment (28-hour forced desynchrony protocol), Thaiss et al. observed significant alterations in microbial community structure and enhanced lipopolysaccharide (LPS) biosynthesis pathways, corresponding with diminished glucose tolerance. The study directly associated microbiome alterations with metabolic dysfunction, although the pronounced circadian manipulation constrains its ecological validity [26].

There is growing evidence that the microbiome and circadian clocks can influence one another. Microbial metabolites influence host tissues to regulate clock gene expression. Butyrate, for example, has been shown to modulate the expression of circadian clock genes, including Per2, in intestinal epithelial cells, suggesting a mechanistic link between microbial metabolites and host circadian regulation [27]. Conversely, alterations in clock genes affect microbial rhythmicity. In a groundbreaking study, mice deficient in the core clock gene Bmal1 exhibited diminished oscillations in microbial diversity and a cessation of rhythmic SCFA production. The same study also involved fecal microbiota transplantation from Bmal1 knockout mice to germ-free mice, demonstrating increased adiposity and metabolic inflexibility in the recipient animals, thereby establishing functional causality. Limitations include the lack of concurrent human validation despite robust mechanistic insights [28].

These interactions significantly affect metabolic homeostasis. Wang et al. conducted a study involving 29 obese adults, revealing that disrupted microbial rhythmicity was associated with diminished insulin sensitivity and altered rhythmicity of circulating amino acid metabolites. Continuous glucose monitoring and stool metagenomics were employed to establish correlations among rhythms; however, causality cannot be determined from this observational design [29]. Time-restricted eating has demonstrated efficacy in restoring microbial rhythmicity in humans. Sutton et al.'s randomized crossover trial of early time-restricted feeding (eTRF) in eight prediabetic men showed that it increased microbial oscillatory amplitude, lowered fasting glucose, and improved insulin sensitivity. However, the small sample size warrants caution [30].

Collectively, these findings point to a model in which the microbiota and the host's circadian system function as an integrated unit. Changes in feeding schedules, sleep patterns, or central circadian signals can affect microbial rhythmicity, thereby influencing metabolic, inflammatory, and GI health. Notably, germ-free animal studies have demonstrated that the absence of microbiota diminishes oscillation of hepatic metabolic genes governing gluconeogenesis and fatty acid metabolism, underscoring that microbial rhythmicity has downstream consequences for hepatic clock function, a relationship with limited translational human data to date [31]. The complex feedback loops between microbial metabolites and host clock genes further support the rationale for chrononutrition and microbiota-targeted chronotherapy as innovative therapeutic approaches.

The gastro-circadian metabolic axis: an integrative framework

**Figure 2:**
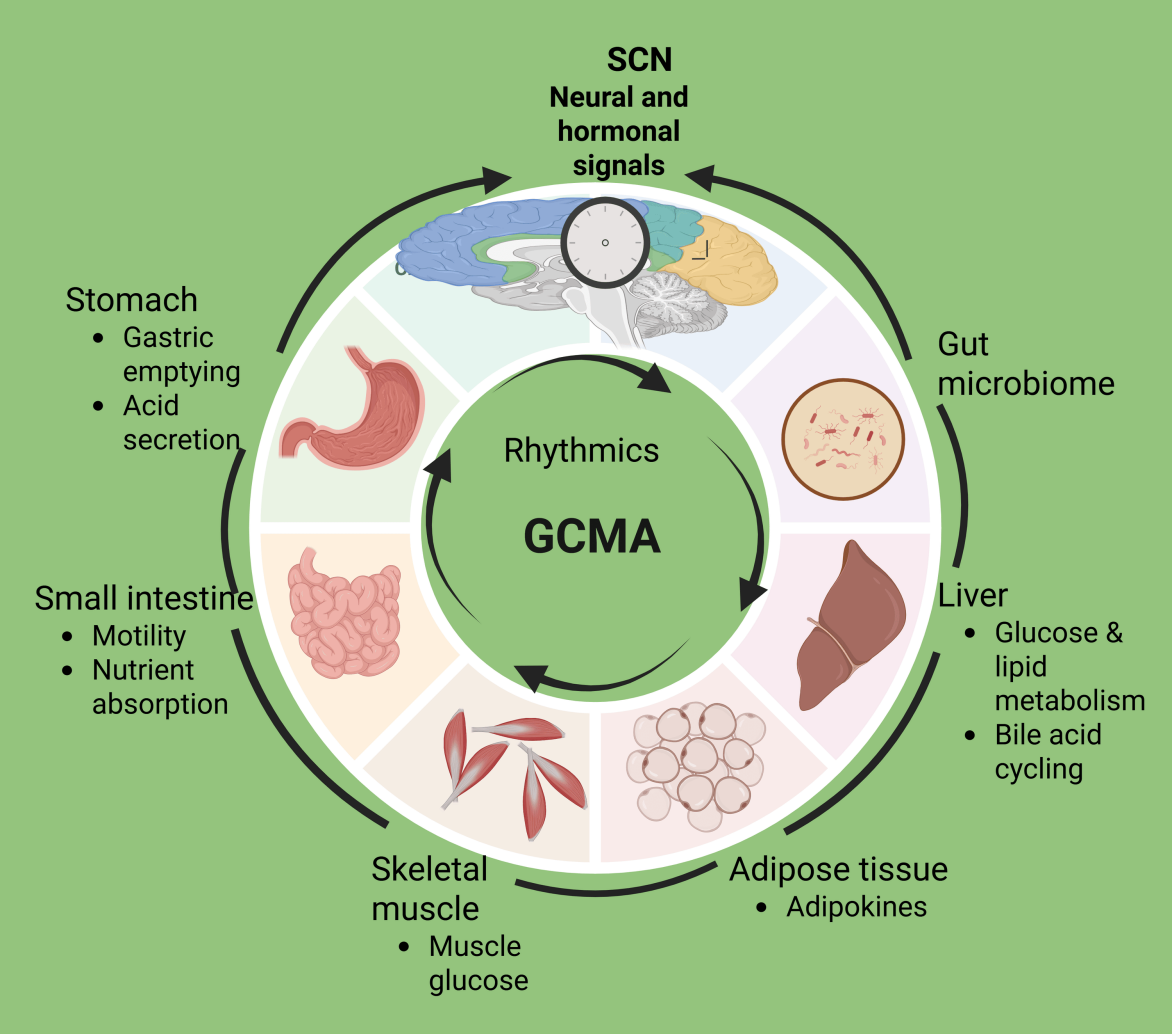
GCMA A conceptual illustration depicting the multilevel interactions among the central circadian clock (SCN), peripheral clocks in GI and metabolic organs, and the gut microbiome. Bidirectional signaling among the stomach, small intestine, colon, liver, pancreas, adipose tissue, and skeletal muscle coordinates 24-hour rhythms in digestion, nutrient absorption, bile acid metabolism, microbial activity, and systemic metabolic regulation. Circadian inputs, including feeding-fasting cycles, neuroendocrine pathways, and microbial metabolites, synchronize peripheral clocks, while chronodisruption alters these pathways and contributes to GI and metabolic disease risk. GI, gastrointestinal; GCMA, gastro-circadian metabolic axis; SCN, suprachiasmatic nucleus Image credit: The figure was created by the authors using BioRender (BioRender.com, Toronto, ON, Canada).

The GI tract, liver, adipose tissue, and skeletal muscle form interconnected metabolic organs whose functions are regulated by circadian rhythms. The GCMA envisions this integration as a series of reciprocal feedback loops connecting gut physiology, microbial metabolism, neuroendocrine pathways, and systemic energy regulation. This framework stresses that digestion and metabolism should not be seen as separate processes, but as parts of a time-structured network that is shaped by behavioral cues, nutrient flow, and endogenous molecular clocks (Figure 2).

Conceptual Model Linking Circadian Rhythms With Gastrointestinal and Metabolic Physiology

Building upon the microbiome and circadian mechanisms discussed in the preceding sections, circadian regulation coordinates digestive functions with environmental and behavioral cues, enabling temporal alignment of nutrient processing during feeding periods and metabolic restoration during fasting phases. Hepatic glucose output, intestinal nutrient transport, and adipose tissue lipolysis exhibit distinct diurnal patterns. Morris et al. conducted a randomized controlled study involving 29 adults to investigate circadian variation in glucose tolerance through three oral glucose tolerance tests administered at 08:00, 14:00, and 20:00 hours. Glucose excursions were markedly elevated in the evening (p<0.001), signifying diminished insulin sensitivity later in the day. Nonetheless, the trial's brief duration and absence of continuous metabolic monitoring restrict its broader applicability [32]. Skeletal muscle also exhibits circadian changes in mitochondrial function. A crossover study conducted by Khatri et al. with 24 healthy men demonstrated that muscle oxidative capacity, evaluated through near-infrared spectroscopy, reached its zenith in the early afternoon, corresponding with enhanced exercise performance. These findings indicate that skeletal muscle clocks affect systemic energy utilization, although the homogeneous male population constrains generalizability [33].

Gut-Liver Axis: Rhythmic Metabolic Exchange

The gut-liver axis is vital to the GCMA. The portal circulation sends enteral nutrients, bile acids, and microbial metabolites directly to the liver, where they interact in a rhythmic sequence. A controlled feeding study by Wehrens et al. involving 18 adults assigned to daytime or nighttime eating schedules showed that eating at night disrupted the normal rhythm of hepatic transcripts that control lipid oxidation. Individuals who ate at 23:00 showed higher postprandial triglyceride levels and lower fasting glucose levels than those who ate during the day, but the small sample size was a limitation [34].

Gut-Brain Axis: Circadian Control of Autonomic and Neuroendocrine Signaling

The gut-brain axis plays a major role in the circadian regulation of digestive processes. A study conducted by Scheer et al. involving 14 healthy adults subjected to forced circadian misalignment exhibited significant disturbances in autonomic balance, characterized by diminished vagal tone and heightened sympathetic activation [35]. These autonomic changes were linked to worse glucose tolerance and higher ratings of gastric discomfort. The study's laboratory conditions were artificial; however, the results demonstrate the impact of circadian signals on GI sensory pathways and metabolic function.

Neuroendocrine mediators further complicate this relationship. Ghrelin, which stimulates hunger, is most elevated just before usual mealtimes and can adjust to feeding schedules independent of caloric load. A study conducted by BaHammam et al. involved 16 adults who altered their regular mealtimes by five hours. Consequently, ghrelin rhythms adjusted within 48 hours, indicating rapid entrainment and underscoring its potential function as a peripheral timekeeper. One limitation is the absence of parallel microbiome data to help explain the observed changes in satiety patterns [36].

Gut-Adipose and Gut-Muscle Metabolic Pathways

Adipose tissue is involved in circadian regulation via the rhythmic secretion of adipokines. In a study of 42 obese individuals, Christou et al. showed that adiponectin had a lower diurnal amplitude than that in lean controls. Reduced adiponectin rhythmicity was associated with elevated hepatic fat accumulation and diminished gut microbial diversity. The cross-sectional design constrains causal inference; however, the findings indicate an interaction between adipose and microbial rhythms [37].

Skeletal muscle has a supporting role in the GCMA. A randomized crossover study involving 93 adults with type 2 diabetes demonstrated that higher-calorie breakfasts and lower-calorie dinners enhanced glycemic control relative to the inverse pattern [38]. Consuming more calories in the morning increased muscular glucose uptake, demonstrating that nutrient utilization is influenced by timing. The study did not evaluate the expression of underlying clock genes, thereby constraining mechanistic understanding [38].

Role in Nutrient Absorption, Energy Utilization, and Hormonal Balance

Circadian coordination across the GCMA enhances nutrient absorption during feeding periods and metabolic repair during fasting. A recent randomized trial involving 90 adults indicated improved postprandial lipid metabolism when meals were consumed earlier in the day [39]. Participants consuming lunch at 13:00 compared to 16:30 demonstrated enhanced triglyceride clearance, a variation ascribed to circadian fluctuations in lipoprotein lipase activity. The study demonstrates that circadian regulation affects energy utilization, although long-term outcomes have yet to be investigated [39].

GCMA dynamics also control hormonal balance. Synchronous oscillations in GLP-1, insulin, and bile acids have been observed in healthy individuals undergoing 24-hour metabolic profiling [40]. Early daytime peaks enabled effective nutrient absorption and glucose clearance. Nonetheless, the study did not categorize participants according to chronotype, a significant determinant of metabolic rhythms [40].

Chronodisruption and gastrointestinal disorders

As outlined in the preceding sections, factors such as sleep disturbances, shift work, social jet lag, irregular meal timing, and nocturnal light exposure can disrupt the coordinated circadian regulation of GI, immune, metabolic, and microbial processes. The downstream consequences of such disruption, namely, impaired gut motility, dysregulated acid secretion, compromised mucosal barrier function, altered bile acid signaling, and dysbiosis, arise through the molecular and neuroendocrine pathways described in the preceding sections. The following subsections examine how these mechanisms manifest clinically across the major GI disease categories.

Functional and Motility Disorders: Gastroesophageal Reflux Disease, Dyspepsia, and Irritable Bowel Syndrome

A fundamental review revealed that bowel motility in healthy individuals exhibits significant circadian variation, characterized by diminished nocturnal activity and pronounced diurnal activity following awakening or meals. Disturbance of these rhythms is associated with symptoms including constipation, diarrhea, or abdominal discomfort, indicative of conditions such as irritable bowel syndrome (IBS) and functional GI disorders [41]. Specifically, altered colonic motor patterns and dysfunctional migrating motor complexes during sleep or shift work were suggested as underlying mechanisms [4,42].

Recent epidemiological evidence substantiates this correlation. A prospective cohort study examining workers on day versus night shifts indicated that consistent night-shift work, especially of extended duration (>3 years) or increased frequency (>7 night shifts/month), was associated with a modest increase in the incidence of IBS compared to day workers (hazard ratio (HR) ~1.21) [42]. The authors found that low-grade systemic inflammation (assessed through an "INFLA score") partially mediated this association, indicating that inflammatory mechanisms play a role in chronodisruption-induced IBS. Nonetheless, limitations include possible residual confounding factors (stress, diet, and lifestyle) and reliance on self-reported symptoms.

Similarly, sleep disorders have been linked to GERD and functional dyspepsia. Individuals experiencing suboptimal sleep quality, nocturnal awakenings, or erratic sleep patterns often report worsening reflux symptoms, irrespective of acid-suppressive therapy [43]. Circadian misalignment likely disrupts gastric acid secretion rhythms, delays gastric emptying, and impairs mucosal protective mechanisms [44].

Inflammatory Bowel Disease and Mucosal Immunity

Chronic circadian disruption may further aggravate immune-mediated intestinal inflammation. A recent synthesis of mechanistic and clinical data indicates that altered clock gene expression in intestinal epithelial and immune cells, resulting from sleep deprivation, irregular light exposure, or shift work, can compromise barrier function, disrupt cytokine release, and redirect mucosal immune responses toward a pro-inflammatory phenotype [41]. Experimental and translational evidence further supports a causal relationship between circadian disruption and intestinal inflammation. In animal models, chronic circadian misalignment has been shown to increase susceptibility to colitis, with heightened inflammatory cytokine expression and more severe histopathological injury [45]. Complementary studies demonstrate that circadian disruption impairs intestinal barrier function, increasing epithelial permeability and facilitating the translocation of luminal antigens, thereby promoting both intestinal and hepatic inflammation [46]. In human studies, altered expression of core circadian clock genes has been identified in intestinal biopsy samples early in the course of inflammatory bowel disease (IBD), suggesting that circadian dysregulation may contribute to disease pathogenesis rather than being solely a secondary phenomenon [47].

Observational data demonstrating that IBD patients with disturbed sleep or irregular daily routines have more severe disease activity and more frequent flares support this hypothesis [41]. Nonetheless, heterogeneity across studies, inconsistent definitions of "chronodisruption," and the absence of prospective trials hinder definitive conclusions.

Metabolic-Hepatic Disorders: Non-alcoholic Fatty Liver Disease/Metabolic Dysfunction-Associated Steatotic Liver Disease

The association between chronodisruption and liver metabolic disease is one of the most extensively studied. A recent systematic review of nine observational studies across diverse occupational groups identified a consistent positive correlation between long-term shift work and metabolic dysfunction-associated steatotic liver disease (MASLD), the newly defined condition that supersedes non-alcoholic fatty liver disease (NAFLD), particularly in individuals with frequent or extended night-shift exposure [48]. Numerous studies indicated that night-shift workers exhibited a greater likelihood of hepatic steatosis compared to daytime workers, with odds ratios increasing in relation to the duration of shift work. Nonetheless, limitations include inaccurate quantification of shift-work exposure, inconsistent definitions of MASLD, insufficient adjustment for confounding variables (diet, BMI, and physical activity), and a cross-sectional design in certain cohorts, which hinders causal inference.

A cross-sectional study of steelworkers found that individuals who had worked shifts for more than 20 years had a 2.3-fold higher likelihood of moderate to severe steatosis than daytime workers [48]. The study identified disrupted melatonin secretion, oxidative stress, mitochondrial dysfunction, and altered bile acid metabolism as potential mechanisms. However, the diagnosis relied on ultrasound instead of liver biopsy, and the metrics for sleep quality were rudimentary (indicating whether sleep was permitted or not, without comprehensive data on sleep duration and quality), both of which were recognized limitations.

Preclinical studies show that disrupting the expression of hepatic clock genes in rodent models leads to impaired lipid metabolism, disrupted glucose homeostasis, and accelerated steatosis, even without dietary excess [49]. These models illustrate that chronodisruption can autonomously induce metabolic liver disease; however, translation to human pathophysiology remains limited.

Gastrointestinal Malignancies

Long-term chronodisruption, especially through shift work and prolonged nocturnal light exposure, has been identified as a potential risk factor for GI cancers (colorectal, gastric, and hepatic) [44,50]. Sleep and circadian disruption may facilitate carcinogenesis through various mechanisms, including compromised DNA repair resulting from disrupted clock gene regulation, heightened oxidative stress, altered cell-cycle control, and persistent inflammation [44,50]. Although epidemiological data remain sparse and somewhat contradictory, the biological rationale is supported by findings of altered clock gene expression in tumor tissues and enhanced tumor proliferation in animal models experiencing chronic clock disruption [51]. However, considerable methodological limitations (heterogeneous exposures, confounding factors such as diet, smoking, alcohol consumption, and the absence of prospective data) inhibit definitive conclusions.

Critical Appraisal and Limitations

Overall, the available data indicate a robust correlation between chronodisruption and GI disorders. However, there are a few limitations to consider. First, numerous human studies depend on self-reported sleep and work history, lacking sufficient characterization of chronotype, light exposure, or meal timing. Second, confounding variables such as dietary composition, physical activity, stress, and socioeconomic factors are frequently inadequately addressed. Third, the majority of studies are either observational or cross-sectional, which restricts causal inference. Fourth, diagnostic criteria for GI diseases differ among studies, diminishing comparability. Notably, interindividual differences in circadian sensitivity (chronotype), genetic variations in clock genes, and environmental factors (light pollution, culture, and work schedules) make generalization even more challenging.

Lastly, the restriction to English-language publications may have introduced language bias and could have resulted in the exclusion of relevant studies or conceptual models published in other languages. Future reviews incorporating multilingual database searches may help determine whether comparable circadian-GI-metabolic frameworks have been proposed in non-English scientific literature.

A summary of key human and animal studies evaluating the relationship between chronodisruption and GI disorders is provided in Table 3.

**Table 3:** Original studies linking chronodisruption to GI disorders GI, gastrointestinal; IBS, irritable bowel syndrome; MMC, migrating motor complex; GERD, gastroesophageal reflux disease; IBD, inflammatory bowel disease; MASLD, metabolic dysfunction-associated steatotic liver disease; OR, odds ratio; HR, hazard ratio

Study (author, year)	Population/model	Chronodisruption exposure	Main findings	Disease focus	Limitations
Konturek et al., 2011 [14]	Physiological studies in healthy humans	Natural circadian variation; disrupted motility rhythms	Demonstrated diurnal bowel motility patterns; rhythm disturbance linked to functional GI symptoms	Functional GI disorders (motility, IBS-like symptoms)	Older physiological data; not an interventional study
Duboc et al., 2020 [4]	Narrative mechanistic review	Sleep disturbance, shift work	Proposed mechanisms linking chronodisruption to abnormal motility, including impaired MMC	IBS/functional motility disorders	Review, not original research
Yao et al., 2025 [44]	Large prospective cohort; day vs. night-shift workers	Night-shift work ≥3 years or >7 shifts/month	Night-shift workers had a higher incidence of IBS (HR ~1.21); inflammation partially mediated the association	IBS	Self-reported symptoms; potential residual confounding
Bishehsari et al, 2025 [42]	Patients with sleep disturbance and reflux/dyspepsia	Poor sleep, irregular sleep-wake patterns	Worsening GERD and dyspepsia symptoms independent of acid suppression	GERD, dyspepsia	Observational; mechanisms inferred but not directly tested
Codoñer-Franch & Gombert, 2018 [43]	Mechanistic review with human mucosal data	Clock gene dysregulation due to circadian disruption	Altered clock gene expression contributes to impaired barrier function and inflammation	IBD	Conceptual model; requires prospective validation
Kim et al., 2022 [48]	Korean steelworker cohort	>20 years shift work	Night-shift workers had OR ~2.3 for moderate-severe steatosis	MASLD	Ultrasound diagnosis; crude sleep metrics; cross-sectional
Ferrell, 2023 [49]	Preclinical rodent studies	Genetic clock disruption; light-at-night	Disrupted hepatic clock expression accelerates steatosis and gut inflammation	MASLD, gut inflammation	Animal model; uncertain human translation
Han et al., 2025 [50]	Review with mechanistic and epidemiological synthesis	Chronic circadian disruption, shift work, light exposure	Clock gene dysregulation implicated in GI tumorigenesis; supports biological plausibility	GI cancers	Limited human prospective cancer data
Sancar et al., 2021 [13]; Fortin et al., 2023 [17]	Animal models and molecular studies	Chronic circadian desynchrony	Altered DNA repair, oxidative stress, and tumor-promoting pathways with circadian disruption	GI malignancies	Preclinical; heterogeneous exposures

Chronotherapy in gastroenterology: translational and clinical evidence

**Figure 3:**
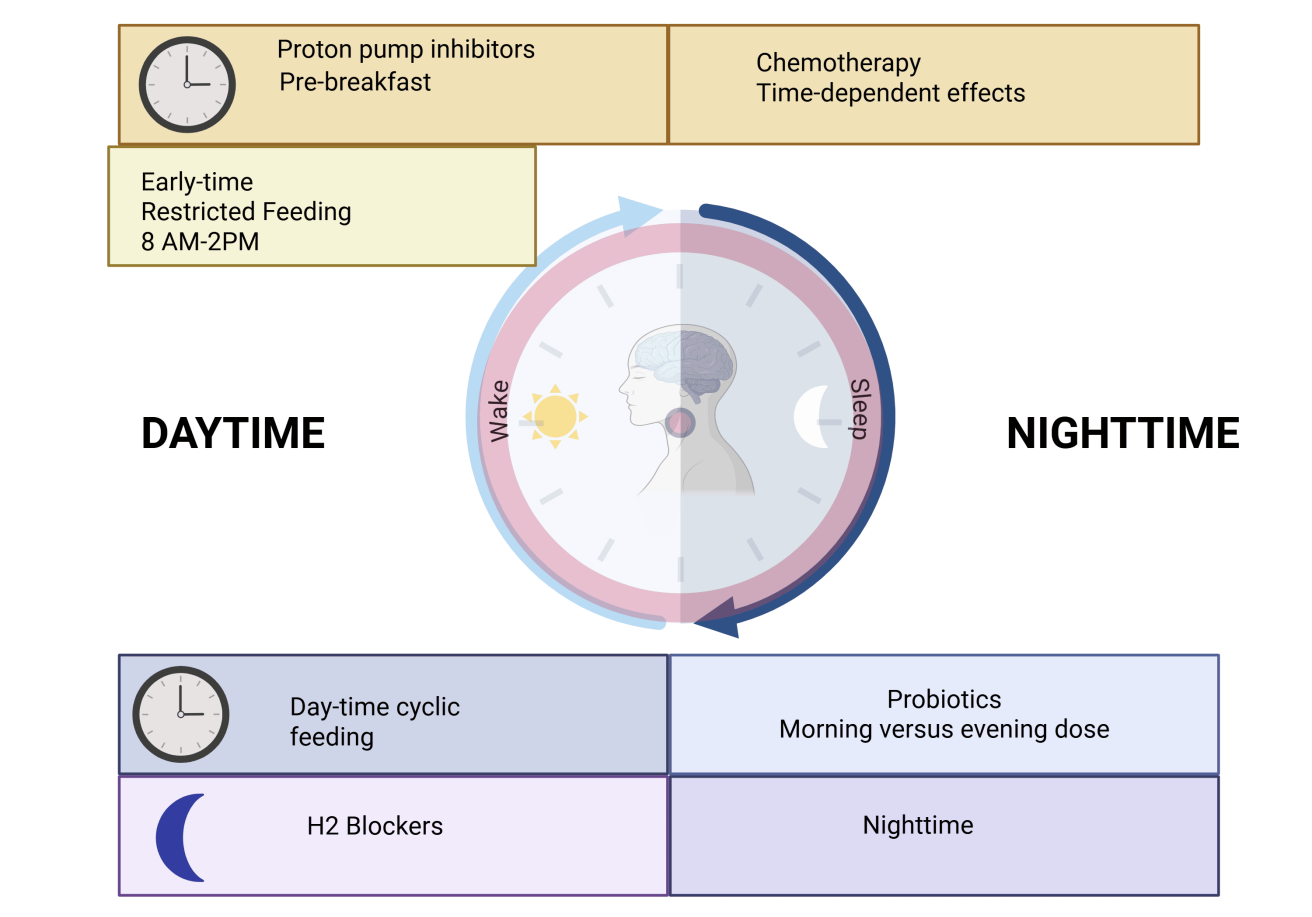
Clinical chronotherapy strategies in gastroenterology A 24-hour timeline illustrating the optimal timing of key gastroenterological interventions. Proton pump inhibitors (PPIs) are most effective before breakfast; eTRF aligns with daytime metabolic activity; chemotherapy may benefit from circadian-based scheduling; probiotics exhibit timing-dependent effects; daytime cyclic feeding supports metabolic alignment; and bedtime H₂ blockers target nocturnal acid secretion. eTRF, early time-restricted feeding; PPIs, proton pump inhibitors Image credit: The figure was created by the authors using BioRender (BioRender.com, Toronto, ON, Canada).

Given the growing evidence linking circadian regulation, microbiome rhythmicity, and GI physiology, chronotherapy aims to optimize therapeutic interventions by aligning treatment strategies with biological rhythms to enhance efficacy, reduce toxicity, and improve clinical outcomes (Figure 3). Although widely explored in oncology, rheumatology, and endocrinology, its application in gastroenterology has expanded only in the past decade. This section reviews key translational and clinical studies across drug therapy, chemotherapy, nutritional interventions, and microbiota-modulating strategies, highlighting both benefits and limitations.

Principles of Chronotherapy

Circadian rhythms affect how drugs are absorbed, distributed, metabolized, and excreted (ADME). There are rhythmic fluctuations in gastric emptying, intestinal transporters, hepatic cytochrome P450 (CYP) activity, bile acid secretion, and renal clearance [52]. Thus, the same medication can have different effects on plasma levels and therapeutic outcomes when administered at different times. The concept also applies to local GI effects. For example, gastric acid secretion is highest at night, colonic motility is highest in the morning, and mucosal permeability changes throughout the day. These characteristics facilitate the judicious scheduling of medications used for acid suppression, motility disorders, inflammatory conditions, and metabolic dysfunction.

Proton Pump Inhibitors and Acid Secretion Rhythms

During active proton pump stimulation, proton pump inhibitors (PPIs) need to be activated in parietal cells. This is why dosing close to the peak stimulus is more effective. A crossover study involving 52 patients with GERD examined the effects of omeprazole 20 mg administered in the morning (30 minutes before breakfast) versus the evening (before dinner). Morning dosing resulted in significantly enhanced 24-hour acid suppression and symptom alleviation, with 60% versus 42% attaining sufficient acid control (p<0.01) [52]. The study's strength is its crossover design; however, its short duration (14 days per arm) limits conclusions concerning long-term effects. Other research demonstrates that nocturnal acid breakthrough often continues even when PPIs are taken in the evening. This suggests that PPIs may work best when taken before the first major meal. Limitations include inconsistent definitions of "nocturnal breakthrough" and differing meal timing among studies. Still, the time-dependent efficacy of PPIs is one of the clearest examples of GI chronotherapy.

Chronotherapy in Chemotherapy for Gastrointestinal Malignancies

Chronomodulated chemotherapy has undergone a comprehensive assessment in colorectal cancer. A significant multicenter randomized trial evaluated chronomodulated infusion against constant-rate infusion of 5-fluorouracil (5-FU), folinic acid, and oxaliplatin in 564 patients with metastatic colorectal cancer. Chronomodulated infusion (oxaliplatin peak at 16:00, 5-FU peak at 04:00) significantly reduced grade 3-4 mucositis and neuropathy in men and improved overall survival (HR 0.79), although the benefits were less consistent in women [53]. Limitations include sex-specific effects and unexamined variations in chronotype. Nevertheless, the trial demonstrated proof-of-concept for synchronizing drug delivery with circadian fluctuations in drug toxicity and tumor vulnerability.

These findings are supported by smaller phase II studies, but they have not been widely adopted because of logistical challenges, limited access to infusion pumps, and the need for personalized circadian profiling. Additionally, chronotherapy appears to be drug-specific, as similar benefits were not observed with chronomodulated irinotecan or cisplatin regimens. These results indicate that pharmacodynamic tailoring is necessary.

Chrononutrition and Meal-Timing Interventions

Meal timing is a significant chronotherapeutic tool. A randomized crossover trial involving eight men with prediabetes compared eTRF (08:00-14:00) with a 12-hour feeding window. eTRF significantly enhanced insulin sensitivity, lowered blood pressure, and increased morning metabolic flexibility, irrespective of weight loss [30]. The limited sample size restricts generalizability; however, the controlled design illustrates that metabolic regulation is significantly time-dependent.

In a 12-week study involving 120 adults with metabolic syndrome, consuming more calories in the morning and fewer at night improved homeostatic model assessment of insulin resistance (HOMA-IR), triglycerides, and postprandial glycemia [54]. Although dietary adherence was self-reported, the findings suggest that synchronizing caloric intake with circadian metabolic peaks enhances glycemic control and may be advantageous for patients with NAFLD/MASLD.

Emerging evidence supports dietary timing as an adjunctive chronotherapeutic strategy in GI inflammatory disorders. In pilot studies of IBD, intermittent fasting coordinated with daytime eating reduced inflammatory markers and improved fatigue scores, although results varied across phenotypes and follow-up was limited [55]. A subsequent randomized controlled study in patients with Crohn's disease demonstrated that aligning food intake with circadian rhythms improved both clinical symptoms and inflammatory markers, providing more controlled support for these early observations [56]. Mechanistic studies indicate that fasting may further reinforce these benefits by influencing the circadian expression of epithelial repair genes and microbial metabolite profiles, although larger and longer trials are needed before clinical recommendations can be made.

Chronotherapy for Inflammatory and Motility Disorders

Glucocorticoids exhibit time-dependent pharmacodynamics because immune activity changes throughout the day. Modified-release prednisone formulations designed for 02:00 release (administered at bedtime) alleviate morning stiffness in rheumatoid arthritis; analogous principles may be relevant in IBD, although direct trials are lacking [57]. A limited mechanistic study involving 19 patients with Ulcerative colitis identified diurnal variation in mucosal cytokine expression, with peak levels of tumor necrosis factor alpha (TNF-α) and interleukin-6 (IL-6) occurring in the early morning hours [58]. These rhythms, although preliminary, indicate possible temporal targets for anti-inflammatory therapy.

Emerging evidence also supports chronotherapy in the administration of immunomodulators for IBD. A clinical study evaluating time-dependent dosing of thiopurines (azathioprine and 6-mercaptopurine) demonstrated that aligning drug administration with circadian immune activity may improve therapeutic efficacy and reduce adverse effects [59]. These findings highlight the potential of circadian-informed scheduling for optimizing immunosuppressive therapy in IBD.

Melatonin, which is released predominantly at night, has been studied as a chronotherapeutic agent for motility disorders. A randomized controlled trial involving 40 patients with functional dyspepsia demonstrated that bedtime melatonin (3 mg nightly) alleviated epigastric pain and fullness more effectively than placebo, potentially through circadian modulation of gastric accommodation [60]. The study was constrained by its brief duration and absence of mechanistic biomarkers, yet it suggests the therapeutic significance of circadian neurohormones.

Probiotics and Time-Dependent Microbiota Modulation

Microbial rhythms indicate that probiotics may exert timing-dependent effects. A randomized trial involving 60 adults assessed Bifidobacterium longum BB536 administered either in the morning or evening, revealing that morning dosing resulted in greater increases in SCFA-producing taxa and enhanced bowel regularity [61]. The proposed mechanism was congruent with morning motility peaks. Nonetheless, the limited sample size and absence of metabolomic profiling restricts mechanistic understanding.

Preclinical research corroborates these results: probiotics administered during the host's active phase yield enhanced immunomodulatory and epithelial barrier effects, presumably due to synchronized microbial colonization rhythms [34]. Human studies remain limited, and dosing recommendations are still being evaluated.

Challenges in Clinical Implementation

Despite the promise of chronotherapy, several challenges must be addressed before widespread clinical implementation. Interindividual variability in chronotype, circadian phase, occupational schedules, and social behaviors complicates the development of universal timing recommendations, as early and late chronotypes may require substantially different medication and meal-timing strategies. Contemporary lifestyles, particularly shift work, nocturnal light exposure, and irregular eating schedules, continue to promote circadian misalignment and may limit the effectiveness of chronotherapeutic interventions in real-world settings. Patient adherence represents an additional barrier, as strict meal-timing protocols, time-restricted feeding regimens, or precisely scheduled medication administration may be difficult to sustain long-term, particularly among individuals with irregular schedules or multiple comorbidities.

Many existing clinical studies are further limited by short durations, small sample sizes, and failure to stratify participants by sex, chronotype, or baseline circadian misalignment, all factors known to influence treatment response. Finally, although circadian assessment technologies such as actigraphy, continuous physiological monitoring, wearable biosensors, and dim-light melatonin onset (DLMO) testing hold promise for circadian phenotyping, their cost, technical complexity, and lack of standardization currently restrict clinical adoption. Future implementation studies should therefore evaluate not only biological efficacy but also patient acceptability, adherence, healthcare resource utilization, and real-world practicality across diverse clinical settings.

Table 4 summarizes key clinical and translational studies evaluating chronotherapy interventions across GI and metabolic disorders [30,38,52,53,55,57,60,61].

**Table 4:** Chronotherapy interventions in gastroenterology: evidence summary PPIs, proton pump inhibitors; GERD, gastroesophageal reflux disease; RCT, randomized controlled trial; 5-FU, 5-fluorouracil; eTRF, early time-restricted feeding; BP, blood pressure; HOMA-IR, homeostatic model assessment of insulin resistance; IBD, inflammatory bowel disease; SCFAs, short-chain fatty acids; TNF-α, tumor necrosis factor alpha; IL-6, interleukin-6; OS, overall survival; HR, hazard ratio; UC, ulcerative colitis; GI, gastrointestinal

Intervention/drug	Rationale for timing	Key evidence (study design & population)	Optimal timing suggested	Main outcomes	Limitations	Ref. no.
eTRF	Aligns caloric intake with daytime metabolic peaks; promotes insulin sensitivity	Randomized crossover trial, 8 men with prediabetes	08:00-14:00 eating window	Improved insulin sensitivity, BP, oxidative stress; weight-independent benefits	Very small sample; short intervention length	[30]
Morning-heavy caloric intake (chrononutrition)	Insulin sensitivity and lipid metabolism highest in morning	RCT in 93 adults with type 2 diabetes: high-calorie breakfast vs high-calorie dinner	Largest meal in the morning	Lower daily hyperglycemia; improved glucose uptake and glycemic control	No clock-gene assessment; adherence self-reported	[38]
PPIs	Proton pumps most active before first major meal; dosing then maximizes PPI activation	Randomized crossover study, 52 GERD patients comparing morning vs evening omeprazole 20 mg	30 minutes before breakfast	Greater 24-h acid suppression; better symptom control; 60% vs. 42% adequate control	Short study duration; inconsistent nocturnal definitions	[52]
Chronomodulated chemotherapy (5-FU, leucovorin, oxaliplatin)	Circadian variation in drug metabolism and toxicity; aligning peaks reduces side effects	Phase III RCT, 564 metastatic colorectal cancer patients comparing chronomodulated vs. conventional infusion	Oxaliplatin peak 16:00; 5-FU peak 04:00	Reduced grade 34 toxicity in men; improved OS in men (HR 0.79)	Sex-specific effects; lacks chronotype data; logistical barriers	[53]
Intermittent fasting (IBD pilot evidence)	Fasting modulates epithelial repair genes and microbial rhythmicity	Case reports and small observational evidence in ulcerative colitis	Daytime-aligned fasting	Reduced inflammatory markers; improved fatigue	Very small, non-controlled studies	[55]
Glucocorticoids (prednisone chronotherapy mechanistic extrapolation)	Immune cytokine peaks early morning; timed release improves anti-inflammatory efficacy	UC mucosal cytokine profiling demonstrates early morning TNF-α/IL-6 surges	Bedtime dose → 02:00 timed release	Potential reduction in inflammatory activity; chronotherapy rationale strong	No GI-specific RCTs; mechanistic but not clinical demonstration	[57]
Melatonin for functional dyspepsia	Modulates circadian GI motility, gastric accommodation, and mucosal defense	RCT, 40 functional dyspepsia patients receiving bedtime melatonin 3 mg	Bedtime dosing	Reduced epigastric pain/fullness; symptom improvement	Short duration; limited mechanistic biomarkers	[60]
Probiotics (timing-dependent administration)	Microbial colonization and SCFA production follow circadian cycles; morning motility peak enhances effect	RCT, 60 adults receiving Bifidobacterium longum BB536 morning vs evening	Morning dosing	↑ SCFA-producing taxa; improved bowel regularity	Modest sample; lacks metabolomic profiling	[61]

Future perspectives and research directions

As our understanding of the GCMA advances, future studies should focus on individualized circadian-based treatment, elucidation of mechanisms, and the feasibility of widespread clinical application. Wearable technology, multi-omics profiling, artificial intelligence (AI), and experimental chronobiology are all advancing several important areas.

One of the most crucial tasks is the development of personalized chronotherapy. Chronotype, sleep patterns, work schedules, and baseline circadian alignment differ significantly among populations, affecting responses to medication timing, meal scheduling, and microbiota-based interventions. A study involving 133 adults employed dim-light melatonin onset (DLMO) and wearable-based rest-activity rhythms to measure circadian phase discrepancies [62]. The study revealed considerable interindividual variability in circadian timing, even among participants with similar sleep-wake schedules, underscoring the inadequacies of self-reported sleep patterns and the need for physiological biomarkers to customize therapy. However, DLMO assessment is resource-intensive, underscoring the need for accessible and scalable circadian biomarkers in clinical practice.

Wearable devices capable of tracking heart rate variability, core body temperature, actigraphy, and continuous glucose monitoring offer new opportunities for real-time circadian profiling. Waqar et al. conducted a study involving 40 participants undergoing continuous physiological monitoring, demonstrating that multimodal wearable biosignals could accurately infer circadian phase with a mean absolute error of less than two hours [63]. Although still in its early stages, this technology may enable chronotype-adjusted medication and nutritional timing in practical clinical settings.

Progress in multi-omics, encompassing metagenomics, transcriptomics, metabolomics, and epigenomics, will elucidate mechanistic connections within the gastro-circadian axis. A multi-omics study of 21 healthy volunteers found that the expression of host genes, microbial metabolites, and circulating lipids changed synchronously, demonstrating that the gut and metabolic tissues function together in a rhythmic manner [64]. The limited sample size and controlled setting restrict generalizability; however, the study demonstrates proof of concept for integrative circadian systems biology.

AI and machine learning could help determine optimal medication timing, identify circadian phenotypes, and model rhythmic interactions among organ systems [65]. Pilot studies employing machine learning to forecast glycemic rhythms or microbiota diurnal variation underscore AI's emerging role in chronomedicine; however, larger and more diverse datasets are needed.

Finally, preventive gastroenterology might incorporate circadian hygiene, regular sleep, timed light exposure, and structured meal timing as modifiable risk factors for GERD, IBS, MASLD, and IBD. Large, long-term studies will be needed to establish causality and develop practical guidelines. These directions will help advance proof-of-concept chronotherapy toward precision gastro-circadian medicine with real-world clinical impact.

## Conclusions

The GCMA offers a cohesive framework for comprehending how rhythmic coordination among the GI tract, microbiota, liver, adipose tissue, skeletal muscle, and neuroendocrine pathways regulates digestive and metabolic homeostasis. Research involving humans and animals indicates that circadian regulation is essential for nutrient absorption, energy expenditure, immune equilibrium, and epithelial integrity. When these rhythms are disturbed, due to shift work, irregular sleep, inconsistent eating habits, or extended light exposure at night, susceptibilities arise across a range of GI disorders, including GERD, IBS, MASLD, IBD, and GI malignancies. Mechanistic studies underscore the significance of altered clock gene expression, compromised barrier function, dysbiosis, and misaligned metabolic pathways as pivotal mediators of disease risk.

Chronotherapy presents a promising translational approach to mitigate these vulnerabilities. The timing of medications, nutraceuticals, and feeding schedules can significantly affect therapeutic outcomes, with increasing evidence in acid-related disorders, metabolic diseases, and cancer therapies. Nonetheless, interindividual variability in chronotype and circadian phase highlights the necessity for personalized, biomarker-driven methodologies. Future research should integrate wearable circadian monitoring, multi-omics profiling, and computational modeling to enhance time-based interventions. By synchronizing clinical practice with biological time, gastroenterology can advance toward precision chronomedicine focused on enhancing outcomes and preventing disease.
